# Data-driven modelling of group formation in the fission–fusion dynamics of Bechstein’s bats

**DOI:** 10.1098/rsif.2022.0170

**Published:** 2022-05-04

**Authors:** Nicolas Perony, Gerald Kerth, Frank Schweitzer

**Affiliations:** ^1^ Chair of Systems Design, ETH Zurich, Weinbergstrasse 56/58, 8092 Zurich, Switzerland; ^2^ Applied Zoology and Nature Conservation, University of Greifswald, Loitzer Strasse 26, 17489 Greifswald, Germany

**Keywords:** Bechstein’s bat, decision making, agent-based modelling, fission–fusion dynamics

## Abstract

Communal roosting in Bechstein’s bat colonies is characterized by the formation of several groups that use different day roosts and that regularly dissolve and re-merge (fission–fusion dynamics). Analysing data from two colonies of different sizes over many years, we find that (i) the number of days that bats stay in the same roost before changing follows an exponential distribution that is independent of the colony size and (ii) the number and size of groups that bats formed for roosting depend on the size of the colony, such that above a critical colony size two to six groups of different sizes are formed. To model these two observations, we propose an agent-based model in which agents make their decisions about roosts based on both random and social influences. For the latter, they copy the roost preference of another agent which models the transfer of the respective information. Our model is able to reproduce both the distribution of stay length in the same roost and the emergence of groups of different sizes dependent on the colony size. Moreover, we are able to predict the critical system size at which the formation of different groups emerges without global coordination. We further comment on dynamics that bridge the roosting decisions on short time scales (less than 1 day) with the social structures observed at long time scales (more than 1 year).

## 1. Introduction

The idea that ‘more is different’ [1] has become a common paradigm to describe a system whose behaviour changes qualitatively when the number of its elements increases. As emphasized by Cavagna & Giardina [2], it is also an interesting perspective from which to look at animal groups. These groups vary widely in size and stability, from small social groups with stable individual composition as in cooperatively breeding mammals to vast aggregations such as kilometre-long fish shoals containing tens of millions of individuals (e.g. [3]). Large variations in group size can also be observed within the same species. Increasing group sizes have been shown to foster division of labour [4,5], transitions from disorder to order [6] or the accuracy of group decisions [7]. Generally, these questions are linked to the topic of optimal group size in animal populations [3].

Here we concentrate on Bechstein’s bat (*Myotis bechsteinii*), a species where the females form maternity colonies of stable individual composition but with high fission–fusion dynamics in summer, during the breeding season (for a detailed species description, see [8]). *Fission–fusion dynamics* refers to the regular splitting into and merging of *groups* within larger social entities such as colonies in the case of bats [9–11]. Previous studies [12] showed that fission–fusion dynamics may result from simple association mechanisms, and often produce right-skewed group size distributions, with many small groups and very few large ones.

Compared with the number of empirical studies on fission–fusion dynamics (e.g. [9,10,13–16]) modelling approaches are less developed. They can be divided into statistical models and generative models. Statistical models, for instance regression models, aim to *infer* from available data the influences that govern the observed dynamics. For example, the frequency of fission and fusion events in reindeer was predicted based on the observed variation in group sizes [17]. An advanced statistical model, the hierarchical Bayesian model, was used to disentangle the influence of other individuals (action, sex) on the individual fission and fusion decisions of spider monkeys [18]. For Bechstein’s bats, pairs of leading and following animals could be identified by means of an advanced statistical inference model [19].

The evaluation of statistical models is usually restricted to comparing the statistical performance of model variants with and without certain influences. This allows one to estimate the impact of these influences on explaining the data, but it gives no insights into the interaction dynamics or decision rules of individuals. This methodological limitation is addressed by generative models, for instance agent-based models. These propose rules, for example, for interactions or decisions, and then test to what extent such assumptions are compatible with an observed behaviour, at either the individual or the systemic level. Agent-based models have been applied already to the roosting behaviour of bats, albeit with a different focus [20–22]. More recently, spatio-temporal patterns resulting from the swarming activity of the Leisler’s bat, *Nyctalus leisleri*, were replicated by means of a swarm algorithm using species and habitat input parameters [23]. Also, observed patterns of travel distance in red-capped mangabeys were reproduced with a model of their seasonal fission–fusion dynamics [24]. The impact of individual compromises between nutritional needs and social interactions on the social network between individuals and a possible irreversible fission was simulated [25] with an agent-based model. These types of models also allow one to test the impact of certain parameters, for example split rates [26,27], on the principal outcome of the fission–fusion dynamics. In most cases, however, the assumed rules cannot be directly matched to available observations. Therefore, agent-based models provide a way to develop hypotheses about unobserved behaviour that can be addressed in subsequent research. We follow this approach in our paper, with a specific focus on Bechstein’s bats.

In bats, most species are social and form groups of variable size and composition, and many of the colonies display fission–fusion behaviour [28]. Female Bechstein’s bats profit from group formation while roosting as they obtain thermoregulatory benefits from clustering [29,30]. The size of the roosting groups depends on the size of the colony [31,32] and can comprise the entire colony or, in the case of a larger colony that splits into several temporary subgroups, a subset of the colony members. From field studies, only data about the sizes and compositions of roosting groups have become available [33], while the mechanisms of the fission–fusion behaviour are largely unknown. Hence, we observe the results of roosting decisions, but have to infer the underlying decision rules.

To better characterize the decisions resulting in the formation of roosting groups, we have to take into account various aspects. Firstly, not all individuals have the same information about possible roosting sites. As Bechstein’s bats forage separately or in pairs at night [34], information available to each individual about the roosting preferences of the other colony members is limited. Field experiments have shown that colony members exchange some information about the location of suitable roosts [35]. The transfer of information in a colony often occurs by forming pairs of leaders and followers, i.e. by bilateral interactions between more and less informed bats [19]. Secondly, social influence is vastly distributed across individuals, reflecting a consistent ranking. Hence, some individuals are more actively and more often involved in the information transfer.

These two issues are not adequately reflected in a notion of ‘collective decision making’, which would suggest a common democratic procedure. On the one hand, all individuals are involved in the decision process and have to pay attention to the decisions of others, as they can only choose roosting sites also shared by other bats. On the other hand, the decisions do not occur in one joint act, but rather based on a sequence of bilateral interactions over the duration of the swarming phase. The result is not one unanimous outcome, not even one majority-driven decision, but most often a decision in favour of different roosting groups of various sizes and at various places [36,37].

In conclusion, roosting decisions follow a daily cycle. During their nightly foraging bats are alone or in pairs and distributed over a large area [34]. At dawn, bats return to the roosting area not all at the same time and have to decide about the roost where they will stay together with other colony members for most of the following day. At dusk, the bats leave their communal day roosts again and the roosting groups dissolve for nightly foraging. Then, this cycle starts over again. The existing literature [38–40] suggests that the decision mechanisms involved in the choice of a communal roosting site may be self-organized. In this paper, we describe such a self-organized decision-making process in Bechstein’s bats. Our modelling approach builds on two previous empirical studies. Kerth *et al.* [32] analysed the social structure of two colonies of Bechstein’s bats. It was shown that Bechstein’s bats, which do not necessarily have high socio-cognitive abilities, develop multi-layer social structures, notably persistent long-term network communities that emerge from roosting associations. Baigger *et al.* [41] discussed the possibility that these social structures might allow colonies of Bechstein’s bats to collectively withstand adverse events such as a population crash.

In the current work, we set out to explore the link between the individual behaviour, i.e. the roost switching, and the emergence of roosting groups of different sizes. To gain empirical insights, we analyse data on the daily roosting behaviour of individuals from two colonies (see §2.1 and Kerth *et al.* [32] for a full description of the data). These data report on the size of daily roosting groups. But the rules that bats follow to make these decisions about their communal day roosts are largely unknown. Therefore, in this paper we propose such rules which take individual preferences and the information transfer between conspecifics into account. These rules are then applied to develop an agent-based model.

In addition, we focus on the *group sizes* that result from such decision dynamics. The question of how groups form and how their size is regulated is still a very topical one, as accurate studies on the temporal dynamics of an animal group are rarely paired with theoretical justifications derived from robust models [42]. The study and characterization of group sizes are an even larger challenge when the animals undergo a fission–fusion dynamics, as in the case of Bechstein’s bats.

Our main interest is to explain how the formation of roosting groups is influenced by the size of the colonies. If ‘more is different’, we should expect the emergence of specific collective behaviour once colonies have reached a critical size. As we report in the empirical findings, the formation of roosting groups of different sizes dependent on the size of the colony is such an emergent phenomenon. We demonstrate that this transition in the behaviour of the colony can be well explained by our model. But we also show that our model is able to reproduce the distribution of durations that bats spend in the same roost before switching to another roost.

Finally, in this paper we also investigate how the formation of groups as part of the fission–fusion dynamics can be related to the emergence of long-term social structures, such as the existence of communities in social networks of large colonies of Bechstein’s bats [32].

## 2. Methods and data

### 2.1. Available data and subsequent measures

For our model validation, we have data available for two different colonies of Bechstein bats (*Myotis bechsteinii*), a larger one denoted by GB2 with 34–46 individuals and a smaller one denoted by BS with 11–18 individuals [32,41]. Both colonies were observed over many years, from 2004 to 2010 for GB2 and from 2004 to 2008 for BS. All bats in both colonies were individually marked with PIT-tags in their first year of life [8], i.e. they can be identified by these tags over years.

Although Bechstein’s bats forage separately or in pairs during the night, they have to roost together during the day to benefit from social thermoregulation [29,30]. Specifically, they form roosting groups that occupy a ‘bat box’ for some days, but then have to change their box because of the need to avoid parasites that accumulate in the boxes [43] and to find optimal roosting temperatures that are weather dependent [44]. One to six of such roosting groups per colony are formed, and their composition can alter every day. These groups can choose from about 150 bat boxes that were placed in the home range of the two colonies [32]. Only about 50 different boxes (out of 150 available in both colonies, together) are occupied by the groups in each season, and members of different colonies do not roost together. Each of the occupied boxes is equipped with an antenna that is connected to an automatic PIT-tag reader that stores PIT-tag numbers, times and dates of each bat entering the box. This way, from 2004 onwards, for the breeding season between April and September, we have daily data about the presence of individual bats in the respective box.

To formalize the information available from the data, we first introduce three different *time scales*. The longest time scale, *y*, is measured in *years*, or seasons. One season consists of about 200 days, during which information about the roosting behaviour of the colony becomes available. This is the time scale at which long-lasting social structures of the colony become visible, such as communities [32]. We will come back to this in §3.3.

The intermediate time scale, *t*, is measured in *days*, i.e. it is also a discrete scale. On this time scale, the fission–fusion dynamics becomes important. Bats form groups for communal roosting; however, these groups are not stable over a long time and dissolve mostly over 1–2 days. The fusion dynamics refers to the merging of multiple groups into a smaller number of larger groups or even into one group, while the fission dynamics refers to an increase in daily roosting groups. On time scale *t* bats decide about their (daily) roost, for which we have information available. The automatic reading resulted in 6655 individual roosting records for BS and 13 845 for GB2. About 97% of the tagged bats passing the antenna in the box entrance could be identified [35].

This registration allows us to subsequently calculate *pairwise roosting associations* for each colony. If *r* ∈ {1, …, *m*} is the discrete number of the box, then *r*_*i*_(*t*) tells us that bat *i* has roosted in box *r* at day *t*. The Kronecker delta *δ*_*ij*_(*t*) then indicates whether two individual bats *i* and *j* have roosted together on that particular day *t*. *δ*_*ij*_(*t*) = 1 if *r*_*i*_(*t*) = *r*_*j*_(*t*), and *δ*_*ij*_(*t*) = 0 otherwise. Aggregating over time for a fixed pair of individuals *i*, *j* tells us how often these two bats have roosted together. If the latter is normalized to the number of days both of these individuals have been observed in the area, it yields the *I*_*ij*_ index [31].

The shortest time scale, *τ*, is much shorter than 1 day and could be measured, for example, in *minutes*. In comparison with the time scale *t*, we can treat this time scale *τ* as (quasi) *continuous*. This is the time scale at which bats *exchange information* about suitable roost sites and *decide* where to roost. We see this communal roosting as the outcome of *group decisions*, for which we can observe the *result* (on the time scale *t*), but do not know the *rules* which generate the observed outcome on the time scale *τ*. To infer a set of possible decision rules that are compatible with this outcome is precisely the aim of our paper. This requires us to model the so-called *swarming phase* more explicitly, during which bats exchange the above information. The swarming phase describes the aggregation of bats that fly around a box at dawn before they eventually use it as a day roost [45]. During this swarming phase, the bats presumably make their decisions about where to communally roost. Before we come to this, we need to take a closer look at the characteristic features of the roosting data, obtained at time scale *t*.

### 2.2. Distribution of duration of stay in a box

**Figure 1.**
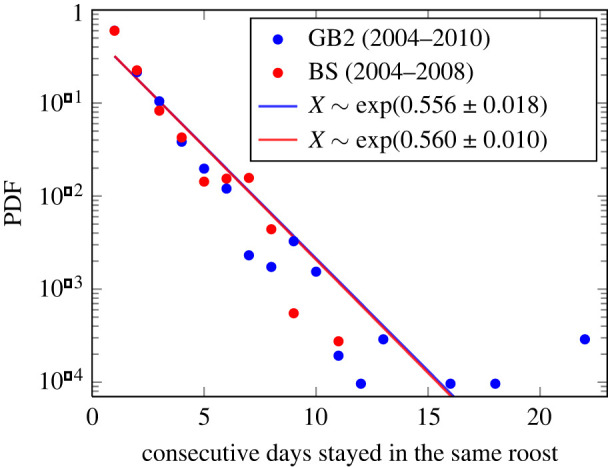
Probability density function (PDF) of the duration of stay for a bat in a given box. The solid lines are fits for both colonies to exponential distributions, with the scale and its 95% confidence interval indicated in the legend.

As we have already mentioned, Bechstein bats have an incentive to switch boxes. Hence, the first question is about their average roosting duration in a given box. To calculate this duration *T*, measured in days, we have to compare, for each bat, their daily roosting locations on consecutive days. This results in a time series of values of *T*_*i*_ for each individual bat. To characterize the colony, we have to determine the distribution *P*(*T*) from the histogram of all *T* values for all bats from the same colony. The result is shown in figure 1. We find, for both colonies, the same exponential distribution *P*(*T*) ∝ e^−*αT*^ with almost the same values of *α*. The statistical details are given in appendix A.

It can be noted that the distribution of consecutive days spent roosting in the same box does not differ for the two colonies of very different sizes, GB2 being about twice as large as BS. This indicates that biological reasons that are largely independent of total colony size, such as parasite infestation in the roost [43] or the roosts’ micro-climate in relation to weather conditions [44], determine the duration of use of the box. In both colonies, we observed that bats changed boxes on average about every 2 days, because the mean period is the inverse of the distribution’s rate *α*. This is in line with values found in previous studies on Bechstein’s bats [31,32,46] and in maternity colonies of other species of forest-dwelling bats (e.g. [16,47]).

### 2.3. Distribution of group sizes

**Figure 2.**
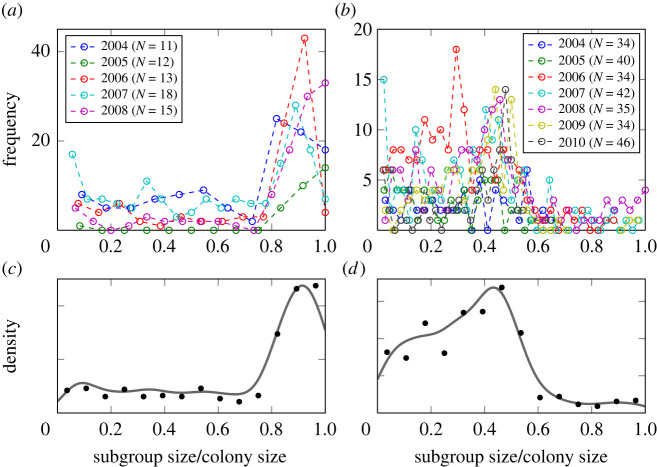
Distribution of relative group sizes *x*_*k*_ in colony BS (*a*,*c*) and colony GB2 (*b*,*d*). Measures are presented for both colonies during all years of the study (*a*,*b*) and aggregated over all years (*c*,*d*). The legends in the top panels indicate the size of each colony in a given year. The aggregated density values (*c*,*d*, black dots) were smoothed using a Gaussian kernel density estimate (solid thick lines) to give a visual indication of the shape of the distribution.

In a second step, we focus on the size of the groups that roost together in one box. Both the number and the maximal size of these groups depend on the size of the colony, which is very different for GB2 and BS. Hence, we have to distinguish between three different levels:

— *N*(*y*) is the size of the *colony*, which can vary from year to year, as figure 2 shows, but is assumed to be fixed for a given year *y* because of the high individual stability of the colonies and the very low mortality of the bats during summer [48].
— Because of the fission–fusion dynamics, each colony is composed of *groups* of different sizes, *n*_*k*_(*t*), where *k* is a group index and *n*_*k*_(*t*) is the size of the group *k* at a particular day *t*. The boundary condition $N=\sum_{k=1}^{K(t)}n_{k}(t)$ has to be fulfilled for each day. The total number of groups, *K*(*t*), is not a constant, but can vary on a daily scale. For comparison of colonies with different sizes, we introduce the *relative group size*, *x*_*k*_(*t*) = *n*_*k*_(*t*)/*N*, with $\sum_{k}x_{k}(t)=1$.
— On the third level, we have *individuals* *i* with *i* = 1, …, *N* that compose the different groups. The composition of the groups can also vary day by day. Sometimes a group can only consist of a single individual that roosts alone and at other times all individuals may form a single group of the size of the colony; i.e. *x*_*k*_(*t*) can vary between 1/*N* and 1 in the extreme cases, which also impacts the total number of groups per day, *K*(*t*).

In figure 2, we calculate, for each colony separately, how often relative group sizes *x*_*k*_(*t*) were observed in a given year. To highlight the differences, we have then calculated from these frequencies, aggregated over all years, the distribution *P*(*x*) for the two colonies.

Comparing the two distributions *P*(*x*), we already note that they are clearly different. The small colony BS displayed a very cohesive behaviour, i.e. all individuals mostly roosted together. The large colony GB2, on the other hand, showed the formation of groups of a size smaller than or approximately equal to one-half of the colony size. Only on very few occasions did the whole colony roost together. There are two main observations from figure 2:

(i) In their roost choice Bechstein’s bats indeed have to take the decisions of others into account, otherwise the formation of groups and the coordinated roosting behaviour would not be observed. This confirms previous findings from empirical studies [36,37].
(ii) Comparing the small and the large colonies, we argue that there is a critical colony size *N*_crit_ above which the formation of more than one roosting group per colony becomes very likely. The graphs indicate that this critical size is approximately 18, because in the smaller colony all members most often roost together in a single group whereas in the larger colony multiple groups of one-third to one-half of colony size are most often formed.

### 2.4. The need to model social influence

In the following, we develop an agent-based model that aims to reproduce the two previously mentioned empirical observations: (i) the distribution of consecutive days of staying in the same box (figure 1) and (ii) the size of the groups that roost together, dependent on the colony size (figure 2).

To start with the exponential distribution of durations *T*, we know that such a distribution can be obtained by assuming a simple Poisson process for the bats’ interactions. Specifically, we could consider that agents every day change from their previously occupied roost to another roost with a fixed probability *α*, and then they opt for another box on the next day. Thus, the chance that they stay at the same roost will decay as 1 − *α*.

One could argue that this decision to leave depends on the available roost sites in the vicinity. As flying long distances is energetically costly for broad-winged bat species (e.g. [49]), Bechstein’s bats may prefer to fly short distances when switching to another box, thereby minimizing their energy expenditure. However, in Bechstein’s bats the foraging area of a bat is typically much larger than its roosting area and thus roost-switching distances may not be relevant to its box choice process [50]. Testing for this effect, we found no effect of the previously occupied box on the next occupied box in terms of flying distance between boxes. The statistical details are again given in appendix A. This insight then lends evidence to the assumption that bats choose *randomly*, with respect to distance, among the available roost boxes. Hence, one could assume that the whole process of leaving a box and choosing another box can be modelled as a random process, where the fixed probability *α* decides *when* to leave.

With such an assumption, we correctly reproduce the distribution *P*(*T*) observed in figure 1, independent of the system size. But, as a consequence of this assumption, we *also* get an exponential distribution of *group sizes*, because agents find themselves together only at *random*. There is evidence for such an exponential distribution of group sizes for *other systems* [12,51–54], but *not* for *our* system of Bechstein’s bats, where the distribution of group sizes is very different from an exponential distribution. As figure 2 shows, in our case the distribution is *not* right-skewed as is the case for an exponential distribution. Further, our distribution also *changes* based on the system size, i.e. the number of bats.

Hence, from these considerations we can conclude that another mechanism is needed in the model to correctly account for the way in which bats communicate about their roosting intention, and form these groups. Therefore, in addition to the *random influence* already assumed for reproducing the exponential distribution of durations, *P*(*T*), we have to add *social influence*. Only this will allow agents to copy the roosting intention (or preference) of other agents, as needed to form groups.

### 2.5. Modelling the swarming phase

As already argued, social influence is exerted at a time scale *τ* shorter than the scale *t*, specifically during the *swarming phase* (at dawn), in which Bechstein’s bats aggregate in flying around potential roosts with the opportunity of signalling their preferences for certain day roosts to other colony members. We assume that, when the swarming phase starts, each agent has a roosting preference *r*_*i*_(*τ*), where *r* ∈ {1, …, *m*} is the identity (number) of the preferred box *r*. The start value for *r*_*i*_(*τ*) is the roost number from the last day. This preference is not fixed, but can change during the swarming phase. In our model, we assume that this dynamics is governed by two different processes: (i) a *random change*, which is modelled again by a Poisson process with a rate *λ* (equal for all agents), and (ii) the *social influence*, which causes an agent *i* to change its roost preference *r*_*i*_ to the *r*_*j*_ of another agent *j* at a rate *γ*.

So, basically these two processes compete: agent *i* picks a new preferred roost either *randomly* or takes the roost preference of other bats into account. The latter means that agent *i* *amplifies* the preference *r*_*j*_ for a given box by *copying* the respective decision from agent *j*. Which of these processes dominates depends on the ratio *λ*/*γ*, which will be determined later during the model *calibration*.

In order to decide when a roosting preference *r*_*i*_(*τ*) is finalized, i.e. does not change further, we could set an arbitrary time after which the swarming phase is finished. However, this would introduce a strict cut-off in the model that can hardly be justified. It further increases the influence of noise on the dynamics, because it is rather arbitrary as to which preferences agents have at a fixed point in time. To avoid such artefacts, we model a progressive decision process in which agents switch one by one to the decided state at a rate *ξ*. We can set *ξ* = 1 for simplicity because both *λ* and *γ* are defined relative to *ξ*. Implementing the decision process in this way allows rich dynamics in which agents finalize their preferences at different times. This allows agents that have already decided about their roost to still influence agents that have not yet decided where to roost.

## 3. Results

### 3.1. Model calibration

To obtain results, we need to calibrate the two parameters introduced: (i) the rate *λ* at which agents *randomly change* their preferences for a roost site during the swarming phase and (ii) the rate *γ* at which agents copy the preferences of other agents. For this calibration, we use the empirical finding *P*(*T*) of figure 1 that demonstrates the outcome of the *combined processes* which jointly determine when agents change their current roost.

Our model generates for each agent a sequence of roost sites *r*_*i*_(*t*) used on consecutive days. From this time series, we can determine the sequence of durations ${\hat{T}}_{i}$ that agent *i* stays in a given box before changing to another box. We deem our model correct if it is able to reproduce the empirical finding *P*(*T*), i.e. if the model-generated distribution $P(\hat{T})$ of durations matches the observations. This implies three requirements: (i) $P(\hat{T})$ has to be an exponential distribution, which is ensured because we have modelled the random change of preferences as a Poisson process; (ii) the characteristic parameter $\hat{\alpha }$ obtained from $P(\hat{T})$ has to match the empirical value *α* = 0.56; and (iii) as an additional constraint, we need to make sure that the model-generated distribution $P(\hat{T})$ is also independent of the system size *N*.

These three requirements can be achieved by adjusting the model parameters *λ* and *γ* such that the match between model and empirics is as good as possible. Specifically, the following two errors have to be *minimized*:

3.1 $$E_{1}=\frac{1}{N}\sum_{N=N_{1}}^{N_{2}}|\alpha_{\text{out}}(N)-\alpha |$$

and

3.2 $$E_{2}=\sqrt{\frac{1}{N_{2}-N_{1}}\sum_{N=N_{1}}^{N_{2}}{[\alpha_{\text{out}}(N)-\left\langle \alpha_{\text{out}}(N)\right\rangle ]}^{2}}=\text{std}(\alpha_{\text{out}}).$$

*E*_1_ measures the difference between $\alpha_{\text{out}}(N)$, the model-generated decay value of the exponential distribution, and the empirical value *α*, which should be as small as possible. The model output $\alpha_{\text{out}}(N)$ depends on the colony size *N*, which we have varied in discrete steps between $\left\{N_{1}\ldots N_{2}\right\}$. Practically, we have chosen *N*_1_ = 10 and *N*_2_ = 50 because these are the typical minimum and maximum colony sizes, respectively [28]. For each value of *N*, we ran 10 000 simulations of the model, hence $\alpha_{\text{out}}(N)$ already gives the average over 10 000 simulations.

*E*_2_ is the standard deviation of the distribution of all $\alpha_{\text{out}}(N)$ obtained in the range *N* ∈ {*N*_1_ … *N*_2_}. This error should be minimized during the calibration because we want the model output to be independent of the system size *N*. $\left\langle \alpha_{\text{out}}(N)\right\rangle$ is the mean value of $\alpha_{\text{out}}(N)$.

**Figure 3.**
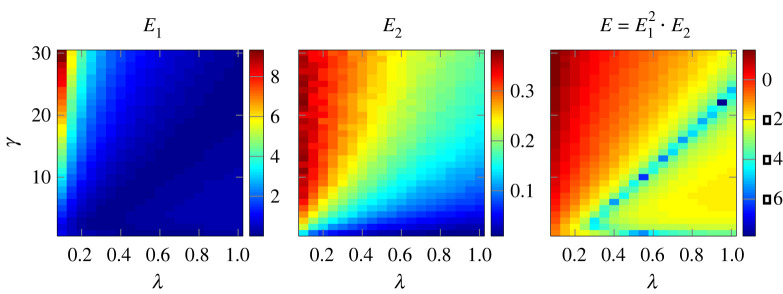
Fitting of the random choice parameter *λ* (*x*-axis) and the social influence parameter *γ* (*y*-axis) by minimization of the model’s total error *E*. *E*_1_ represents the exponential fit error with regard to the experimental data and E_2_ the *model stability error*, i.e. the variation of the mean consecutive roosting period with regard to the system size *n* (number of agents), with *n* ∈ {10 … 50}. For all plots, the error should be minimized, thus less (dark blue) is better; scales are logarithmic.

To obtain the pair {*λ*, *γ*} that best fits the experimental data, we minimized the product of the squared error of the exponential fit by the size-related error, $E=E_{1}^{2}\cdot E_{2}$. Plots of *E*_1_, *E*_2_ and *E* are shown in figure 3. We found a minimum of *E* for *λ* = 0.95 and *γ* = 22.

### 3.2. Distribution of group sizes

The agent-based model outlined above shall now allow us to reproduce, and to understand, the second empirical finding, namely the distribution of the sizes of groups that roost together, dependent on the size of the colony, as shown in figure 2. Specifically, the small study colony roosted mostly as a single group, while the larger colony roosted in several groups, with the largest roosting group comprising mostly about half of the colony.

**Figure 4.**
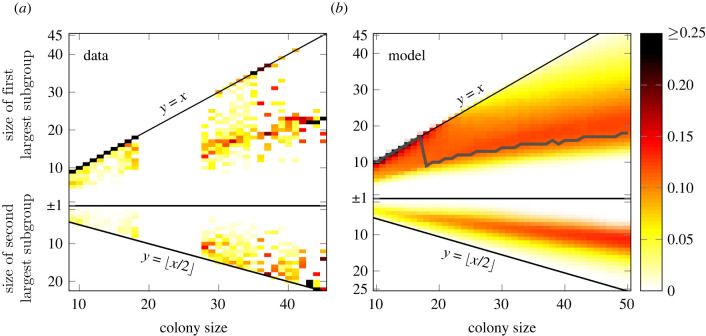
Empirical group sizes (*a*) and modelled group sizes (*b*) as a function of colony size. Colours represent the probability of an individual belonging to a group of a certain size. The top part of both figures shows the size of the largest group; the bottom part the size of the second largest group. The line *y* = *x* indicates that there is only one group of the size of the colony. If a second largest group exists, its size can be maximally *y* = *x*/2, as indicated in the lower part. The grey line in (*b*) represents the most common largest group size in our simulation results.

However, we do not have observations about the *transition* from one to several groups. Therefore, in a first step we merge the information about the group sizes of the two colonies. Figure 4*a* shows the complete empirical data. The *x*-axis displays the size of the colony, *N*, varying as before between *N*_1_ = 10 and *N*_2_ = 50. The *y*-axis displays compressed information about the size of the groups. The diagonal *y* = *x* shows the *maximum size* of a group for a given size of the colony. If all individuals belong to only *one* group, then we should find the observed group size very close to this diagonal. This is indeed the case, as figure 4*a* shows, but only as long as *N* is below 20. Specifically, we plot the probability of an individual to be part of a group of a given size in terms of a colour code. The darker the colour, the larger this probability.

For colony sizes *N* between 18 and 26, we do not have any empirical data. But for *N* > 26, we see that the group size quite often differs from the colony size, i.e. most individuals are found in groups of sizes much below the diagonal *y* = *x*. This clearly indicates the formation of groups inside the colony. To better understand how the colony splits into groups of *different sizes*, we have plotted in figure 4*a* the size of the largest group, *n*_1_(*t*), in the upper part and the size of the second largest group, *n*_2_(*t*), in the lower part (we have used the group index *k* to rank groups according to their size *n*_*k*_*,* such that *k* = 1 refers to the largest and *k* = 2 to the second largest group).

Obviously, the maximum size of *n*_2_(*t*) is bound to *y* = *x*/2, otherwise it would be the largest group. We see that the empirical sizes of the second largest group are close to this line, but not too close. That means that colonies do not split precisely into two groups of size *N*/2, which would also not be realistic. Instead, we note the formation of the second largest group with sizes *n*_2_(*t*) comparable to about *N*/3. The sizes of the largest group, *n*_1_(*t*), shown in the upper part of figure 4*a*, are comparable to *N*/2. This means that, if other groups exist, they can only be of rather small sizes, summing up to about *N*/6.

Figure 4*b* shows the same diagram with the results of our calibrated agent-based model. We note that simulations were also performed for colony sizes where no empirical data were available (18–26 individuals). The grey line in figure 4*b* shows the most common size obtained for *n*_1_(*t*) in our simulations. This curve displays a sharp drop for a colony size of *N* = 18 individuals. Hence, it marks the transition from a regime where only *one group* of the size of the colony is observed to a regime with *multiple groups* of different sizes. The *critical size* of the *colony* for this transition is *N*_crit_ = 18.

We note the very good agreement of our model results with the empirical observations. Despite the fact that we only used two parameters, *λ*, *γ,* to describe the roosting decision of agents, the model is able to reproduce the findings about group sizes dependent on colony size. This leads us to the conclusion that the underlying dynamics for the agents capture the systemic behaviour to a high degree. In particular, the simulations allow us to project the dynamics of the colonies to the unobserved cases. In this way, we identified the *critical system size* *N*_crit_ = 18 at which the bifurcation in the systemic behaviour, i.e. the transition from a single to a multi-group regime, occurs. We further elaborate on this fact in the Discussion.

### 3.3. Including the roosting history

So far, the two rates *λ* and *γ* have been the same for all agents. This is justified for *λ* because random influences are considered. For *γ*, however, one would expect that the *social influence* between any two agents also depends on their previous experience together. Hence, instead of an overall rate of social influence, we introduce a pair-specific social influence *γ*_*ij*_(*t*) that depends on the joint history of agents *i* and *j*. If these agents have roosted together at a particular day *t*, this should increase their mutual social influence by an amount Δ*γ*. If, on the other hand, these agents never roost together again, this mutual social influence *γ*_*ij*_(*t*) should decay over time at a rate ɛ. This can be expressed by the following discrete dynamics:

3.3 $$\gamma_{ij}(t+1)=\frac{1}{\varepsilon }[\gamma_{ij}(t)+\delta_{ij}(t)\Delta \gamma ].$$

We recall that *δ*_*ij*_(*t*) is the Kronecker delta, which equals 1 whenever *r*_*i*_(*t*) = *r*_*j*_(*t*), i.e. when agents *i* and *j* roost together at day *t*, and zero otherwise.

The dynamics of equation (3.3) follows the idea of *reinforcement learning*, because a previous joint experience in roosting increases the mutual social influence, which in turn increases the future chances that either agent *i* or *j* copies the roost preference of the other agent. Hence, it describes the formation of social bonds between agents that could also impact the long-term social structures, as discussed below. Mutual social influence that is not maintained, however, will decay over time.

*γ*_*ij*_(0) denotes the start value of the mutual social influence. To set this value, we account for the fact that social influence between individuals of the same group is larger than between individuals of different groups. Hence, initially we create two groups of equal size *N*/2. Within these two groups, we set *γ*_*ij*_(0) = 0.55 for all individuals in the same group, and between these two groups we set *γ*_*ij*_(0) = 0.45. Further, we choose Δ*γ* = 0.05 and 1/ɛ = 0.95. The latter describes an exponential decay of the mutual influence, *γ*_*ij*_(*t*) = *γ*_*ij*_(0)exp{[(1/ɛ) − 1] *t*}, if *i* and *j* never share a roost. By choosing the parameters this way, we ensure that 0 < *γ*_*ij*_(*t*) < 1 for any *i* and *j*, regardless of their roosting history.

With this, the individual social influence exerted on agent *i* is no longer a constant *γ*, but an individual parameter, $\gamma_{i}(t)=\sum_{\;j}\gamma_{ij}(t)$, that changes over time and considerably depends on the individual roosting history of an agent. Hence, the dynamics for *γ*_*ij*_(*t*) bridges two time scales: the time scale *t* at which agents roost together and the time scale *y* at which long-lasting social structures of the colonies, such as communities in the social network [32], become visible and important. Ideally, we should observe the emergence of such communities when bridging these two time scales.

**Figure 5.**
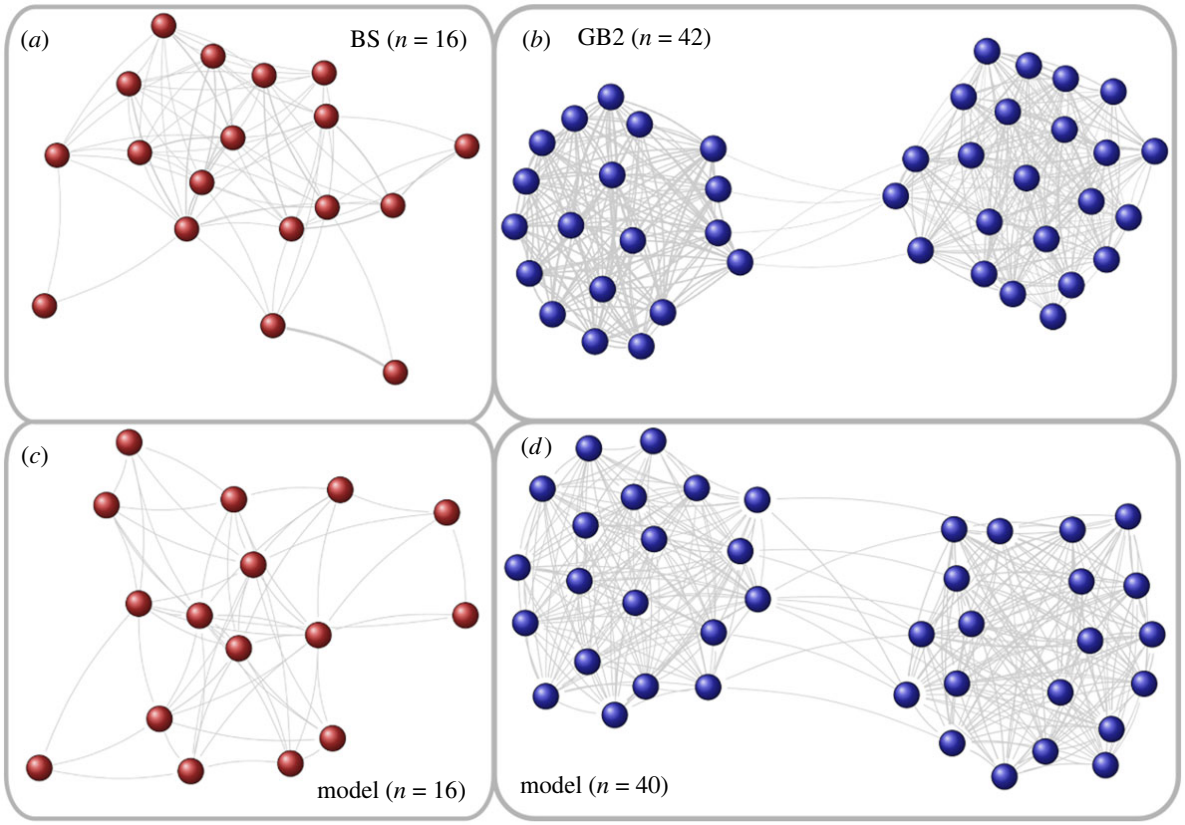
Social network of long-term roosting associations for small (*a*,*c*) and large (*b*,*d*) colonies. (*a*,*b*) Empirical data from the colonies BS (*a*) and GB2 (*b*) in the year 2007. These figures are modified from Kerth *et al.* [32]. (*c*,*d*) Model-generated social network with dynamic *γ*_*ij*_(*t*) after t=200 days. For clarity, in all networks only the strong ties, with a weight larger than the mean value of the weight distribution, are shown.

Figure 5 illustrates the impact of this model modification. Figure 5*a,b* shows the social network of the two colonies as extracted from roost association data on the time scale *y* of a whole year (season) [32]. While the smaller colony, BS, does not display any community structure, the larger colony, GB2, clearly has two communities. Figure 5*c*,*d* shows the social network as obtained from our model using the adaptive *γ*_*ij*_(*t*). We note that these structures are observed *after* the respective *γ*_*ij*_(*t*) have been relaxed to some quasi-stationary values, i.e. after t=200 days.

The interesting finding here is not so much the existence of the two communities in colony GB2. We recall that, in our initial conditions, we have already introduced two groups of size *N*/2 and have argued about slightly different initial values *γ*_*ij*_(0) for agents within the same group versus agents in different groups. With this in mind, we cannot claim the *emergence* of two communities. However, we note the very stable community structure: because this colony GB2 is, with *N* = 42, well above the calculated critical colony size, *N*_crit_ = 18, small initial differences in the social influences, expressed by *γ*_*ij*_(0), not only persisted over a long time but were amplified by the daily fission–fusion dynamics. This eventually resulted in the appearance of two separate network communities on the seasonal time scale.

Another more interesting finding is the *disappearance* of the same group structure when modelling the smaller colony BS. This colony had a size *N* = 16 *below* the critical colony size, *N*_crit_ = 18. Hence, even with the same set-up for *γ*_*ij*_(0), the daily fission–fusion dynamics was not able to sustain the induced two groups to transform them into stable communities. Thus, on the seasonal time scale we obtain with our model the emergence of a single community that is identical to the colony. This lends strong evidence to the assumed dynamics for *γ*_*ij*_(*t*), and to the agent-based model of roosting behaviour.

## 4. Discussion

In this paper, we studied the roosting dynamics of two colonies of Bechstein’s bats, from both the *empirical* and the *modelling* perspective. Our interest was to better understand the *fission–fusion dynamics* in forming roosting groups. Fission means that one colony forms more than one group, while fusion means that all members of one colony are in the same group. Such groups facilitate communal roosting on a daily scale, but do not form social structures that are stable over a long time; i.e. they are different from long-lasting community structures that can be detected in larger colonies [32]. This makes communal roosting particularly interesting and motivates us to model it. Bats forage at night on their own; nevertheless, they manage to meet in communal roosts in the morning. If they stayed in a cohesive group for both day and night, there would be no need to model group formation.

The main contribution of our paper is in formally establishing the link between a simple, common individual behaviour (roost switching at a given frequency) and an empirically observed collective roosting behaviour [32,41] at different colony sizes. As our model shows, it needs social interactions and information flow between the interacting individuals to lead to communal roosting and, subsequently, to long-term social links among colony members. In the following, we further discuss some implications of our investigations.

### 4.1. Emergent structures: group sizes, roosting durations

With our investigations, we follow a *bottom-up approach*, explaining the *emergence* of the groups from the interactions of the individuals that constitute the colonies. These interactions are described by simple rules that agents follow in making roost decisions; i.e. our modelling assumptions focus on the *micro*, or agent, level and the rules are defined on the shortest time scale, *τ*. We want to reproduce the groups observed on the *macro*, or system, level on the intermediate time scale, *t*.

Methodologically, we argue that our agent-based model is correct if it is able to reproduce the observed macroscopic quantities, which explains their emergence from micro-interactions. More specifically, we can deduce that our rules are compatible with the observed system properties, i.e. the size of the roosting groups. Investigations of how bats make their decisions need more data at higher temporal resolution. Hence, further research is needed to focus on this specific question. But we can clearly state that the rules that successfully describe the emergence of the system properties provide suitable *hypotheses* for the behaviour of biological entities.

What kind of emergent properties can we reproduce? The first one is the exponential distribution of durations *T*, i.e. the time spent at the same roost, before switching to another roost. The quantity *T*_*i*_ is measured for individual bats, but only the aggregation to the system level allows us to determine the distribution *P*(*T*), which follows a very simple form with only one characteristic parameter *α*. Importantly, our empirical analysis shows that this distribution is independent of the colony size *N*, which also was reproduced by our model.

For the second emergent property, namely the formation of groups of different sizes inside a colony, we do have a dependence of the colony size *N*. Specifically, small colonies mostly form one roosting group, i.e. all colony members share the same roost, while the larger colonies mostly split into roosting groups of different sizes. This emergent behaviour was also reproduced by our model.

Hence, our agent-based model is able to reproduce two very different and not directly related phenomena, which depend differently on the system size. This lends further evidence to the model.

### 4.2. Modelling individual decisions

The dynamic phenomenon we are interested in basically follows a *simplified daily rhythm*, already described in §1. It repeats a cycle of (i) nightly foraging alone or in pairs, (ii) a decision-making phase at dawn during which bats decide about the roost where they will stay, and (iii) the formation of groups which roost together. At dusk, groups dissolve and this cycle starts over again.

Our agent-based model of roosting group formation specifically models the decision-making phase occurring at dawn every day before the bats start roosting. This process is modelled at the time scale *τ*. For each agent, the roosting decision is affected by two parameters: *λ* describes random influences, whereas *γ* describes social influences exerted from other bats. We note that, with only the random influence, we would be able to reproduce the distribution *P*(*T*) but not the observed group structure. This leads to the conclusion that the influence coming from the roosting decisions of other bats needs to be explicitly taken into account. Here we assumed that agents simply copy the roost preference of other agents at a rate *γ*. We further considered that agents finalize their roosting decisions at different times, which allows us to capture the influence of agents that have already decided on those that have not decided yet.

Individual decisions balance between two concurrent requirements: the pressure to change roosts, e.g. because previous roosts are contaminated with parasites [43], and the pressure to roost together with other colony members, e.g. for thermoregulatory purposes [29,30,44]. Our model reflects that information transfer between bats about roosts plays an important role. We consider that, during the decision-making phase, one individual may copy this information from another one, with a fixed rate *γ*. This way, our model presents an agent-based approach to a fully decentralized, self-organized group decision process.

The model contains two free parameters, *λ* and *γ*. We determined these two parameters *indirectly*, by simulating the model outcome for the duration of stay in the same roost as before, which is determined by *both* parameters. We then adjusted these two parameters such that (i) the discrepancy between the observed and the modelled distribution *P*(*T*) and (ii) the variance of this distribution were minimized. We note that this *model calibration* does not involve information about the group sizes. Instead, the comparison between observed and simulated group size was used to estimate the model performance, independent of the calibration.

### 4.3. Interactions in small versus large colonies

A major empirical finding of our study was that colonies split differently into roosting groups, dependent on their size. This confirms previous empirical findings on Bechstein’s bats [31]. Small colonies mostly form one group, whereas larger colonies form several groups, the largest one comprising about half of the size of the colony. The question is whether this *transition in the system dynamics* dependent on the system size can be understood as an *emergent phenomenon*; i.e. can this transition be obtained by assuming the *same* interaction rules between agents in large and small systems or does it imply *different* interaction rules or rules in which an explicit size dependence is encoded?

With our model, we demonstrated that this transition indeed is an emerging phenomenon that occurs at a critical system size *N*_crit_. Our simulations allowed us to determine this critical value as *N*_crit_ = 18, which is also in line with observations.

Moreover, our model is able to reproduce the group sizes for systems *both* smaller and larger than *N*_crit_, using the *same interaction rules*. We found that larger colonies form groups such that the largest group comprises about one-half of the colony and the second largest group about one-third of the colony, which is also supported by empirical data.

### 4.4. Short-term versus long-term social structures

The collective behaviours of Bechstein’s bats have to be described on three different time scales. The *decisions* about roosts occur during the swarming phase, on the time scale *τ*, shorter than 1 day. The *roosting in groups* occurs on the time scale *t* measured in days; i.e. every day, the groups formed before dissolve and new groups are formed. The question is how this dynamics relates to other dynamical processes observed in the colony on longer time scales. Specifically, Kerth *et al.* [32] and Baigger *et al.* [41] already reported that the long-term social network of larger colonies of Bechstein’s bats consists of *communities*. These are quite stable social structures that can be detected over years.

To bridge between the short-scale and the long-scale dynamics, we allowed the social influence to evolve over time, on the day time scale *t*. Specifically, we turned the homogeneous parameter, *γ*, equal for all agents, into an individual parameter $\gamma_{i}(t)=\sum_{\;j}\gamma_{ij}(t)$, where *γ*_*ij*_(*t*) describes the mutual social influence of agents *i* and *j* as a result of their common roosting history. For the dynamics of *γ*_*ij*_(*t*), we adopted reinforcement learning, i.e. *γ*_*ij*_(*t*) increases if *i* and *j* roost together and it decreases if they do not.

This dynamics occurs over many days; in this way it couples the system dynamics on day time scales with the long-term behaviour. As a result, we could demonstrate that groups existing on day time scale can translate, over time, into long-term social structures, such as communities, *if* these systems are larger than the critical size *N*_crit_. In systems smaller than *N*_crit_, on the other hand, we could show that even induced group structures cannot be transformed into long-term community structures. This agrees with empirical observations that report the absence of such community structures in small colonies [32,41].

We note that the daily splitting into multiple groups and the duration of stay per roost can be modelled with a relatively simple self-organizing mechanism based on a constant *γ*. But for the formation of long-term stable communities in the larger of the two colonies, we need to introduce an individual memory effect, expressed in *γ*_*i*_(*t*), that allows the bats in the model to keep a record of their previous roosting history.

We conclude that our agent-based model is well posed to capture two different empirical observations in Bechstein’s bats, namely the distribution of stay lengths and the distribution of group sizes, which are not inherently connected. This lends evidence to the assumed rules of interactions because they are able to reproduce these different systemic properties. Comparing our model with other agent-based models of fission–fusion dynamics discussed above, we highlight that none so far has addressed the social and temporal aspects of the fission–fusion dynamics together. Moreover, in comparison with formally advanced, but rather abstract models [26,27] or mere simulation approaches [25], our model outcome can be directly compared with the respective empirical observations in Bechstein’s bats. This was also achieved by the agent-based model of red-capped mangabeys with respect to travel distance patterns [24]. But their model relied on a large number of parameters proxied from field data, whereas our model has the advantage of a simple, yet convincing approach of the empirical observations.

## Data Availability

The code and the data are provided in the electronic supplementary material [56].

## Appendix A

**A.1. Testing for the distribution of roosting durations**

When comparing the distribution of roosting duration periods from one year to another, we found that 60% of the time for BS and 48% of the time for GB2 the distributions were not significantly different (we compared all yearly distributions one with another; colony BS, years 2004–2008; colony GB2, years 2004–2010; two-sample Kolmogorov–Smirnov test with Šidák correction for multiple sampling, *n* = 10 for BS and *n* = 21 for GB2, *p* > 0.05).

Finally, the aggregated distributions of roosting duration periods for each colony did not differ significantly from one another, neither in their exponential fit (figure 1) nor in their observed distribution (colony BS, years 2004–2008: *n* = 3633 roosting periods in total; colony GB2, years 2004–2010: *n* = 10 385; two-sample Kolmogorov–Smirnov test, *p* = 0.63).

Based on these observations, we concluded that the durations of stay in a given roost varied neither within the colonies, with different years of observation (hence, different colony sizes), nor between the two observed colonies (despite them having distinct roosting areas; see for example [55]).

**A.2. Testing for the influence of distances**

For each bat in each year, we randomized the sequence of visited roosts and compared the distribution of flying distances aggregated over a large number of randomizations with the observed distribution (a Monte Carlo simulation of the roosting sequence).

We found that, for 91% of the time, the sequence of distances between consecutively occupied roosts for a bat was not significantly different from what it would be if the bat had visited the same roosts in a random order (colony GB2, years 2005–2010; average number of roosts successively occupied per individual and per year, *n* = 38.8 ± 12.3; non-parametric two-sample Kolmogorov–Smirnov test with Šidák correction for multiple sampling within years, *n* = 231 individuals over all 5 years, *p* > 0.05 for 211 out of 231 series of observations).

Moreover, we identified no effect of the previously occupied roost on the next occupied roost in terms of flying distance between roosting sites. In light of these observations, and in order to adopt a parsimonious approach, we modelled in a first step the roosting behaviour of an individual bat by a zero-order Markov process (or random walk).
